# Osteoprotegerin in breast cancer: beyond bone remodeling

**DOI:** 10.1186/s12943-015-0390-5

**Published:** 2015-06-10

**Authors:** Michael Weichhaus, Stephanie Tsang Mui Chung, Linda Connelly

**Affiliations:** Division of Natural Science and Mathematics, Chaminade University of Honolulu, 3140 Waialae Avenue, Honolulu, HI 96816 USA; Department of Pharmaceutical Sciences, Daniel K. Inouye College of Pharmacy, University of Hawaii at Hilo, Hilo, HI 96720 USA

**Keywords:** Osteoprotegerin, Bone, TNF-related apoptosis inducing ligand (TRAIL), Apoptosis, Angiogenesis, Metastasis

## Abstract

Osteoprotegerin (OPG) is a secreted protein and member of the Tumor Necrosis Factor (TNF) Receptor superfamily. OPG has been well characterized as a regulator of bone metabolism which acts by blocking osteoclast maturation and preventing bone breakdown. Given this role, early studies on OPG in breast cancer focused on the administration of OPG in order to prevent the osteolysis observed with bone metastases. However OPG is also produced by the breast tumor cells themselves. Research focusing on OPG produced by breast tumor cells has revealed actions of OPG which promote tumor progression. *In vitro* studies into the role of OPG produced by breast tumor cells have demonstrated that OPG can block TNF-related apoptosis inducing ligand (TRAIL)-mediated apoptosis. Furthermore, *in vivo* studies show that OPG expression by breast tumors can promote tumor growth and metastasis. In addition it has been shown that OPG stimulates endothelial cell survival and tube formation thus it may indirectly promote breast tumor progression through impacting angiogenesis. This article will present a summary of the data concerning the tumor-promoting effects of OPG in breast cancer.

## Introduction

Osteoprotegerin (OPG) is a secreted protein that acts in the bone to block the maturation of bone resorbing osteoclast cells and thus prevent bone breakdown [1, 2]. This physiological role led to investigation of the use of recombinant OPG as a treatment to prevent the bone breakdown that can be initiated when breast cancers metastasize and grow in the bone. However primary breast tumors also express OPG indicating the potential for OPG to have additional roles in breast tumorigenesis [3, 4]. Significant evidence has now accumulated from cell culture and animal model studies indicating that OPG can promote breast tumor growth and spread. This review will present current work illustrating the tumor promoting effects of OPG in breast cancer. These studies demonstrate that OPG should be considered in a dual light in breast cancer having actions that can both promote breast tumor progression as well as prevent bone destruction.

## Review

### Identification of OPG and characterization as a mediator of bone re-modeling

OPG was initially identified through sequence homology to the tumor necrosis factor (TNF) receptor superfamily. OPG contains the four cysteine-rich domain repeats characteristic of the TNF receptor family but lacks a transmembrane region and is therefore a secreted protein [1, 5, 2]. In addition to the cysteine-rich repeats OPG contains two death domain homologous regions as well as a heparin-binding site [6].

Generation of transgenic mice has allowed elucidation of the major physiological role of OPG as a regulator of bone metabolism. OPG over-expression results in a lack of mature bone-resorbing osteoclasts and a pronounced increase in bone density known as osteopetrosis [1]. Mice in which the gene for OPG has been knocked down show the opposite phenotype – an increased number of mature osteoclasts and severe bone loss or osteoporosis [7, 8]. These phenotypes combined with *in vitro* osteoclast maturation studies demonstrate that OPG acts as a negative regulator of bone metabolism and blocks osteoclast maturation [1, 2]. Subsequent studies revealed that OPG exerts this effect by acting as a decoy receptor for Receptor Activator of NF-kappaB ligand (RANKL). OPG binds to RANKL preventing its interaction with the receptor RANK and thus blocks stimulation of osteoclast maturation [9, 10].

### OPG and bone metastasis

Given the characterization of the role of OPG as a negative regulator of bone metabolism as described above, early studies on OPG and breast cancer focused on the bone microenvironment. Bone is the most common site of breast cancer metastasis. Breast tumors tend to form osteolytic metastases where the tumor is surrounded by osteoclasts which degrade the bone tissue [11-13]. An imbalance of the RANK/RANKL/OPG pathway has been observed in tumors with an up-regulation of RANKL and a down-regulation of OPG [14]. As OPG is known to inhibit osteoclast maturation, studies have looked at the use of recombinant OPG to prevent bone loss related to bone metastasis. Truncated forms of recombinant OPG were developed containing the N-terminal portion of the protein necessary for interaction with RANKL fused to the Fc domain of human IgG. The Fc-OPG construct prevented formation of osteolytic metastatic lesions by MDA-MB-231 human breast cancer cells in nude mice [15] and in ovariectomised nude mice [16]. In addition, a reduction in serum markers of bone resorption was observed in a Phase I study in which Fc-OPG was administered to breast cancer patients [17]. Further testing of this construct was not pursued due to concerns regarding an immune response against endogenous OPG [18]. A monoclonal antibody against RANKL, Denosumab, has instead been developed and approved to prevent skeletal-related events and bone loss in patients with advanced breast cancer [19]. Denosumab is also being tested in a Phase III clinical trial, D-CARE (ClinicalTrials.gov Identifier: NCT01077154), to determine whether it can prevent breast cancer recurrence in the bone or other tissues in women with early-stage breast cancer at high risk of recurrence.

These early studies focused on actions of exogenously added, truncated OPG within the bone microenvironment. However, subsequent studies that are described below demonstrate a more extensive and complex role for OPG in breast cancer.

### OPG expression in breast cancer cells and tissue

One of the first studies to characterize OPG revealed expression in two human breast cancer cell lines – MDA-MB-436 and MCF-7 [5]. Further studies have confirmed the expression of OPG by breast cancer cell lines and tissues. The MCF-7, MDA-MB-231 and T47D human breast cancer cells lines were tested for OPG mRNA expression by RT-PCR alongside 12 primary breast tumor samples [20]. OPG mRNA expression is up-regulated in human breast cancer cell lines and tumor samples. The expression pattern of OPG was examined by immunohistochemistry in 400 invasive breast cancer tissue samples [3]. It was found that 40 % of the invasive breast tumors expressed OPG with expression confined to tumor cells. This finding was validated in a recent study where 175 patient breast tumor samples were analyzed and it was found that 45.9 % of tumors expressed OPG [4]. It should be noted that the expression of OPG by tumor cells is not limited to breast cancer. Expression of OPG has been observed in other cancer cell types including colon, prostate and bladder cancer illustrating the potential for a broader role in tumorigenesis [21].

While there have been no investigations focusing on OPG in normal breast epithelium, OPG knockout mice are able to maintain litters which would suggest that OPG is not required for normal mammary gland development and lactation [8]. It appears that OPG expression is associated with breast tumor formation.

### Regulation of OPG expression in breast cancer

The studies described above demonstrate a basal level of expression of OPG in breast cancer cells and tissue. Further investigation has revealed the potential for modulation of OPG expression levels within breast tumors. Immunohistochemical staining of human breast tumor samples showed that OPG expression correlated with the presence of the Estrogen and Progesterone receptors [22]. However a functional investigation of the link between the Estrogen Receptor (ER) and OPG expression suggests a negative relationship where activation of ER reduces OPG expression. Treatment of the ER-positive MCF-7 breast cancer cell line with 17beta-estradiol inhibited OPG mRNA and protein expression and this effect was reversed by the estrogen receptor antagonist ICI 182,780 [23]. While estrogen appears to down-regulate OPG expression, treatment of MCF-7 and MDA-MB-231 breast cancer cells with Interleukin-1Beta increased OPG mRNA and protein expression [24].

### Interaction between OPG and TNF-related apoptosis inducing ligand (TRAIL)

TNF-related apoptosis inducing ligand (TRAIL) is secreted by normal tissues and preferentially induces apoptosis in tumor cells [25, 26]. The screening of OPG protein with TNF-related ligands led to the discovery that OPG could bind to TRAIL, preventing its interaction with Death Receptors and blocking TRAIL-induced apoptosis of Jurkat cells, a human T-lymphocyte cell line [27]. OPG appears to act as a “decoy” receptor for TRAIL, preventing interaction between TRAIL and the Death Receptors and exerting an anti-apoptotic effect. Several studies demonstrating the OPG-TRAIL interaction have been performed *in vitro* using human breast cancer cell lines. TRAIL-induced apoptosis in MDA-MB-436 and MDA-MB-231 human breast cancer cells could be reduced by the addition of recombinant OPG (10-1000 ng/ml). MDA-MB-436 cells which had been incubated for 72 to 120 h to allow accumulation of endogenous OPG were less susceptible to TRAIL-induced apoptosis. This effect could be reversed by the addition of excess RANKL to compete for OPG binding [3]. This anti-apoptotic effect of OPG was confirmed through the use of OPG siRNA. MDA-MB-231 cells treated with OPG siRNA to knock down OPG expression had a higher rate of apoptosis when treated with TRAIL as compared to control cells [24].

While *in vitro* studies clearly demonstrate effects of the OPG-TRAIL interaction, the relevance of this interaction for breast cancer cells has yet to be demonstrated *in vivo*. MDA-MB-231 human breast cancer cells were transfected to over-express full length OPG. Despite showing a significant increase in OPG expression along with a reduced sensitivity to TRAIL-mediated apoptosis *in vitro*, there was a lack of effect *in vivo*. Two weeks after intra-tibial injection in mice of cells the anti-tumor effect observed with TRAIL treatment was identical between empty vector and OPG over-expressing human breast tumor cells [28].

The role of OPG produced by breast cancer cells may be more complex *in vivo* due to simultaneous presence of TRAIL and RANKL. An *in vitro* binding study demonstrated that OPG binds TRAIL and RANKL with equal affinity [29]. However the effect of OPG on TRAIL-mediated apoptosis in human breast cancer cells can be reversed by the addition of excess RANKL [3, 24]. This presents a potential mechanism whereby OPG could be prevented from impacting TRAIL due to high RANKL levels in the bone microenvironment. The outcome of OPG signaling in the presence of biologically relevant concentrations of both ligands remains to be determined.

### Tumor promoting role of OPG in breast cancer

There are now a number of *in vivo* studies that indicate tumor-promoting effects of OPG on breast cancer cells, potentially via TRAIL-independent mechanisms as described below. MCF-7 human breast cancer cells were engineered to express Parathyroid Hormone allowing them to form osteolytic metastases after intra-tibial injection in nude mice. Administration of truncated recombinant Fc-OPG reduced bone destruction in agreement with previous studies. In contrast, when the MCF-7 cells were transfected to over-express full length OPG and injected into mice tibias there was an increase in osteolysis. The intra-tibial tumors from the cells over-expressing OPG had an increase in cells staining positive for the proliferation marker Ki67. These MCF7 cells over-expressing OPG produced larger tumors compared to control cells after injection into the mammary fat pad although in this context there was no difference in Ki67 staining. The MCF-7 cells over-expressing OPG did not show any alteration in sensitivity to TRAIL treatment *in vitro*, suggesting that there must be additional mechanisms whereby OPG can have a tumor-promoting effect [30]. The contrasting results in this study may be related to whether OPG is in the truncated or full length form or whether it is exogenously added or produced by breast cancer cells. In a subsequent study MDA-MB-435 human breast cancer cells were treated with a conditional replicating adenovirus to express the truncated OPG-Fc protein. These cells showed a reduction in bone tumor formation and decrease in osteoclast numbers compared with control adenovirus cells suggesting that production of protein from tumor cells themselves can still have a protective effect in the bone [31]. However given the controversy surrounding the origin of the MDA-MB-435 cell line, these results may not represent a breast cancer specific effect [32].

Another *in vivo* study suggests that the tumor-promoting effects of the Estrogen-Related-Receptor-Alpha (ERRα) in breast cancer may be mediated in part via OPG. ERRα expression in bone metastasizing MDA-MB-231 human breast cancer cell derivatives led to an increased expression of OPG. In agreement with this experimental observation, the analysis of 251 patient samples showed that OPG levels were significantly higher in ERRα-positive tumors compared with ERRα-negative tumors. High ERRα and high OPG expression in breast tumor tissue from patients is associated with a greater increase in risk of recurrence than high expression of ERRα only. An increase in tumor growth was observed when the ERRα expressing cells were implanted in the mouse mammary fat pad however a reduction in osteolysis was observed when these cells formed bone metastases after intravenous injection in mice [33]. Therefore an increase in OPG expression by breast cancer cells may contribute to tumor growth at the primary tumor site while inhibiting the destruction and consequent growth in the bone.

This possibility that OPG can protect the bone microenvironment from tumor growth while promoting growth in other tissues is also evident in a recent study. MDA-MB-231 human breast cancer cells were stably transfected to express OPG and injected into the tibias of nude mice. Over-expression of OPG by injected breast cancer cells protected bone from destruction and inhibited growth of the tumors within the bone. However breast cancer cells over-expressing OPG persisted to grow in the space outside of the bone. In addition, OPG over-expressing breast tumor cells injected into the bone had an increased tendency to metastasize to lungs [34].

Despite the accumulating evidence that OPG produced by breast cancer cells may promote tumor growth in sites outside of the bone microenvironment, the mechanism behind this effect is unclear. We have recently performed a study using MDA-MB-231 human breast cancer cells transfected with sh- or siRNA to knock down OPG in a chick embryo *in vivo* model of breast cancer metastasis. Knockdown of OPG expression reduced metastasis formation in chick tissues both from a primary tumor and after direct introduction of cells by intravenous injection. Our *in vitro* mechanistic studies show that OPG knockdown cells express lower levels of Matrix Metalloproteinase-2 and have a reduced ability to invade through a collagen matrix. This would suggest that OPG can exert an autocrine effect and promote breast cancer cell invasion and metastasis [35].

The findings of these *in vivo* studies suggest that while the interaction between OPG and the bone microenvironment can lead to an inhibition of bone tumor growth, OPG can promote breast tumor formation and growth at other sites.

### OPG and endothelial cells

While OPG produced by breast cancer cells may exert effects directly on breast tumor cells it appears that OPG’s interaction with other cells in the tumor microenvironment may also promote breast tumorigenesis. In particular, several studies have reported an impact of OPG on endothelial cells suggesting the potential role of OPG to regulate angiogenesis.

Human umbilical vein endothelial cells (HUVECs) and Human Dermal Microvascular Endothelial cells (HuDMECs) both express OPG. Treatment of endothelial cells with recombinant OPG (100 ng/ml) promotes cell survival and formation of cord-like structures. Analysis of human breast tumor tissues showed that OPG is expressed by endothelial cells in breast tumor tissue but not in normal surrounding tissue. There is a strong positive correlation between endothelial OPG expression and tumor grade, and a strong negative correlation between endothelial OPG expression and ER expression [36]. Furthermore, while endothelial cells are not sensitive to TRAIL-mediated apoptosis, conditioned medium from endothelial cells protected MDA-MB-436 human breast cancer cells from TRAIL. This effect was lost when OPG was removed from the conditioned medium with antibody treatment. This would suggest that OPG from endothelial cells can promote breast cancer cell survival. A subsequent study further demonstrated the crosstalk between breast tumor and endothelial cells related to OPG. T47D human breast cancer cells were co-cultured directly with HuDMECs for 72 h. There was an increase in OPG protein expression in the co-culture supernatant as compared with HuDMECs cultured alone. Isolation of each cell type and real-time quantitative PCR showed the co-culture stimulated the expression of OPG mRNA in the HuDMECs but not in the T47D. This contact mediated induction of OPG expression in HuDMECs by the breast cancer cells was prevented by the presence of an Integrin α_v_β_3_ antibody in the co-culture medium [37]. This would suggest that Integrin α_v_β_3_ signaling can promote OPG expression. This study also demonstrated that OPG (10 ng/ml) promoted tube formation by HuDMECs confirming the potential for OPG to exert pro-angiogenic effects.

Several studies have investigated the mechanism whereby OPG could promote angiogenic behavior of endothelial cells. HuDMECs treated with OPG were elongated with extensive actin networks compared to untreated cells. OPG (0.5-2ug/ml) led to cytoskeletal reorganization and cell spreading; this may be mediated by Focal Adhesion Kinase (FAK) as phosphorylation of FAK (Y397) was induced by treatment with OPG. OPG-mediated HuDMECs proliferation was blocked by the Erk 1/2 inhibitors U0126 and PD98059 while OPG-induced cell migration and cord-like structure formation was blocked by the Src inhibitor PP1 [38]. The ability of OPG to impact these cell signaling pathways associated with adhesion, migration and proliferation was confirmed in a later study performed with endothelial colony forming cells and HUVECs. OPG treatment (0.4nM) of endothelial cells led to a transient increase in adhesion associated with phosphorylation of FAK. OPG also promoted phosphorylation of Erk1/2 and Akt [39]. Treatment of the cells with enzymes that degraded heparin and chondroitin sulphate led to a reduction in OPG-stimulated migration and chemotaxis hinting that the heparin binding domain of OPG may be involved. However the impact of OPG on migration, chemotaxis and tubulogenesis was maintained when a truncated form of OPG (1–194) was used indicating the heparin binding domain was not necessary for pro-angiogenic effects.

The cumulative findings of these endothelial cell studies demonstrate the potential for OPG to exert tumor-promoting effects by stimulating angiogenesis in addition to the direct effects of OPG on breast cancer cells.

### OPG expression and breast cancer prognosis

A few studies have investigated links between OPG and breast cancer prognosis although conflicting findings have been reported. Real-time quantitative PCR was used to analyze the expression of OPG mRNA in 127 primary human breast cancer tissues. It was found patients with higher expression levels of OPG had significantly poorer overall survival as compared with patients who had lower OPG expression levels [40]. However, in a subsequent study where 175 human breast tumor samples were analyzed, no link was found between OPG expression and skeletal disease-free survival, disease-free survival or overall survival [4]. Another study which analyzed publicly available microarray data for 295 breast cancer patients reported that high expression of OPG correlated with longer overall and disease free survival [41]. It is possible that variation in findings relates to differing effects of OPG dependent on breast cancer subtype. An analysis of publicly available microarray data looking at OPG in relation to human breast cancer subtype found that expression of OPG correlates with a better prognosis in ER positive breast cancer while a statistically significant difference was not observed in ER negative breast cancer [42]. Interestingly, among the tumors that expressed high levels of OPG there was a lower fraction of ER positive cases and a higher fraction of triple negative breast cancers. In agreement with this, we have analyzed OPG mRNA expression in publicly available microarray data for human breast cancer cell lines and found that cell lines from the basal subtype have higher levels of OPG expression than luminal subtype cells [35]. Further investigation is required to determine the links between OPG expression, prognosis and breast cancer subtype.

### OPG gene amplification and polymorphisms

In addition to the expression of OPG, changes in the OPG gene have been reported in breast cancer. We analyzed the TCGA-2013 human breast invasive carcinoma data set, through the cBioPortal website, and found that OPG gene copy gain occurred in 182 out of 934 tumors in the set (19.4 %). The presence of an OPG copy number gain is a significant predictor of decreased overall survival in this cohort [35]. The occurrence of single nucleotide polymorphisms (SNPs) in the OPG gene in association with breast cancer has been examined. In one study, it has been reported that breast cancer patients were more likely to have the minor allele C genotype for the OPG SNP rs3102735 as compared to healthy controls [43]. The presence of this variant was associated with a 1.5-fold increased risk of breast cancer. Breast cancer patients with invasive tumors were more likely to have the major allele G associated with the OPG SNP rs2073618 as compared with patients with non-invasive tumors. Another study reported the C allele of the OPG SNP rs2073618 and T allele of the OPG SNP rs2073617 occurred more frequently in breast cancer patients [44]. The impact of these polymorphisms on OPG expression or function in breast cancer requires further investigation.

## Conclusions

**Fig. 1:**
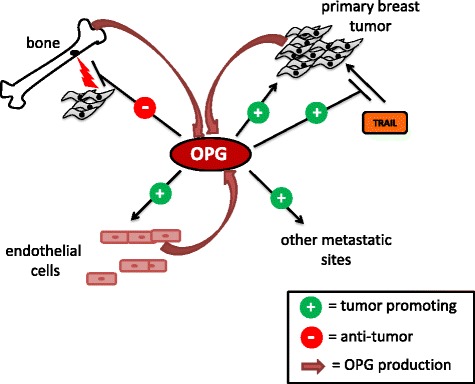
Summary of reported sources and effects of OPG in breast cancer. OPG is produced by bone cells, endothelial cells and breast tumor cells. OPG can exert tumor promoting effects on the primary breast tumor through blocking the action of TRAIL and through direct effects on tumor cells. OPG can promote growth of endothelial cells and tubule formation. OPG promotes formation of metastatic tumors at sites out with bone. OPG production in the bone microenvironment can block the bone destruction associated with growth of metastatic breast tumors

Since its initial discovery and characterization, evidence has been accumulating that OPG plays a role in breast tumorigenesis. The reported actions of OPG in breast cancer are summarized in Fig. 1. It appears that the effects of OPG in breast cancer may vary depending on site of action. OPG is present in the bone during breast tumor metastasis and OPG is also expressed by breast tumor cells and endothelial cells within breast tumors. In the bone microenvironment OPG’s interaction with bone cells can prevent the osteolysis (bone breakdown) associated with breast cancer metastases. However the interaction of OPG with breast cancer cells themselves can lead to tumor growth and spread. This may occur via the ability of OPG to block the induction of apoptosis by TRAIL or through other novel mechanisms. Furthermore, the interaction between RANKL and OPG remains to be investigated in the context of the primary breast tumor. OPG further extends its impact in the breast tumor microenvironment via stimulating growth of endothelial cells and promotion of tubule formation. The effects of OPG may be related to source of OPG with different outcomes resulting from exogenous addition versus production by breast cancer cells themselves. Differing effects have also been observed with truncated OPG versus full length OPG suggesting that the different domains within the protein mediate multiple types of actions within the breast tumor.

While OPG clearly has potential to modulate breast tumor growth and progression, further investigation is required to fully determine the mechanism of effect and overall outcomes of the different types of interactions. The data available currently would suggest that OPG can no longer be considered solely in the bone microenvironment in breast cancer and caution must be exercised in the development of systemic treatment strategies aimed at increasing OPG levels. Localized delivery of OPG to the bone may be more appropriate to prevent osteolysis associated with metastatic breast cancer. Additional studies are required to determine whether strategies to block OPG signaling would be effective in blocking the growth and progression of primary breast tumors.

## Abbreviations

OPG: Osteoprotegerin
TNF: Tumor Necrosis Factor
TRAIL: TNF-related apoptosis inducing ligand
RANKL: Receptor Activator of NF-kappaB ligand
ER: Estrogen Receptor
ERRα: Estrogen-Related-Receptor-Alpha
HUVECs: Human umbilical vein endothelial cells
HuDMECs: Human Dermal Microvascular Endothelial cells
FAK: Focal Adhesion Kinase
SNPs: Single nucleotide polymorphisms
